# Fiber Laser-Generated Silver-109 Nanoparticles for Laser Desorption/Ionization Mass Spectrometry of Illicit Drugs

**DOI:** 10.1021/jasms.3c00454

**Published:** 2024-05-06

**Authors:** Zuzanna Krupa, Joanna Nizioł

**Affiliations:** † Doctoral School of Engineering and Technical Sciences at the Rzeszów University of Technology, 8 Powstańców Warszawy Avenue, 35-959 Rzeszów, Poland; ‡ Rzeszów University of Technology, Faculty of Chemistry, 6 Powstan ´ców Warszawy Avenue, 35-959 Rzeszów, Poland

**Keywords:** cannabinoids, opioids, monoisotopic silver nanoparticles, mass spectrometry quantification

## Abstract

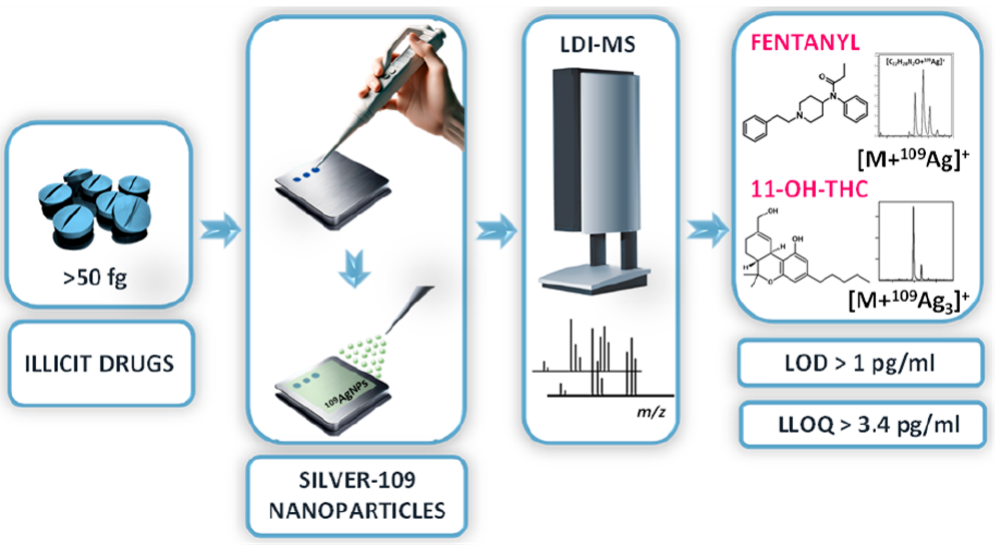

Cannabinoids and opioids are the most prominently used drugs in the world, with fentanyl being the main cause of drug overdose-related deaths. Monitoring drug use in groups as well as in individuals is an important forensic concern. Analytical methods, such as mass spectrometry (MS), have been found most useful for the identification of drug abuse on a small and large scale. Pulsed fiber laser 2D galvoscanner laser-generated nanomaterial (PFL 2D GS LGN) was obtained from monoisotopic silver-109. Nanomaterial was used for laser desorption/ionization mass spectrometry of selected illicit drug standards with standard high-resolution reflectron-based time-of-flight MALDI apparatus. Δ^9^-THC, 11-OH-THC, 11-COOH-THC, fentanyl, codeine, 6-monoacetylmorphine (6-MAM), heroin, tramadol, and methadone were chosen as test compounds. Illicit drugs were tested in a concentration range from 100 μg/mL to 10 pg/mL, equating to 50 μg to 50 fg per measurement spot. For all analyzed compounds, identification and quantification by silver-109-assisted laser desorption/ionization (LDI) MS was possible, with uncommon [M + ^109^Ag_3_]^+^ and [M – H]^+^ ions present for certain structures. The results of the quantitative analysis of drugs using silver-109 PFL 2D GS LGN for LDI MS are presented. Laser-generated NPs are proven to be useful for the analysis of selected drugs, with exceptionally good results for fentanyl monitoring in a broad range of concentrations.

## 1 Introduction

According to the United Nations Office on Drugs and Crime (UNODC) global estimates of the prevalence of drug use in 2021, the use of opioids, including opiates and prescription opioids, was determined to be 1.18% of the world population (0.72, 1.49),^1^ which makes it over 3 times higher than in 2017.^2^ The term opioids refers to exogenous compounds, both natural and synthetic, which bind to opioid receptors. Natural opiates, i.e. codeine, are obtained from the poppy plant as alkaloids constituting a substance known as opium.^3^ The remaining opioids analyzed in this paper are either of synthetic or semisynthetic origin and metabolites. Among them, fentanyl continues to be the drug of choice due to being relatively easy to produce but most importantly having a high potency, 100 times higher than morphine.^4^ The epidemic of fentanyl-induced overdose deaths in the United States started around the year 2015 with 9580 deaths connected to this cause and reached its first peak in 2017, amounting to 28 466 deaths. Since then, as of 2021, the amount of overdose-induced deaths caused by synthetic opioids, primarily fentanyl, increased roughly 2.5 times, with 70 601 deaths.^5,6^ In contrast, the European Monitoring Centre for Drugs and Drug Addiction (EMCDDA) reports 49 confirmed deaths linked to fentanyl, with a minimum estimate of 137 deaths overall in Europe in the year 2021.^7^

Opioid use is the second most prevalent illicit drug use reported by UNODC, the first one being cannabis at 4.27% (3.08, 5.38). The most distinctive class of compounds found in cannabis plants are cannabinoids.^8^ A part of this group, Δ^9^-tetrahydrocannabinol (Δ^9^-THC), is considered as the main compound contributing to the psychoactive effect of cannabis use. THC is metabolized in the human body, resulting in the formation of 11-hydroxy-Δ^9^-THC (11-OH-THC) and 11-nor-9-carboxy-Δ^9^-THC (11-COOH-THC).^9^

Mass spectrometry (MS) coupled with liquid (LC) or gas chromatography (GC) is currently the most widespread analytical technique for illicit drug identification. Several research studies focus on employing LC-MS in estimating illicit drug use in a population rather than in an individual. One of those approaches is sewage forensics, which focuses on analyzing samples of wastewater from certain living areas.^10−12^ For fast identification of illicit drugs in a specific suspect of drug use, approaches such as fluorescence-based lateral flow assay for THC detection in the saliva are used.^13^ Another more conclusive technique is electrochemical detection, with a broad spectrum of solutions for various drugs, giving the possibility of testing oral fluids^14^ or seized powders.^15^

**Table 1:** List of Identified Ions with Mean Signal Intensity and Signal-to-Noise Ratios of Analyzed Compounds at 100 μg/mL Concentration

compound	ion type	mean intensity	S/N
11-OH-THC	[M + H]^+^	1024	7.7
	[M + Na]^+^	3107	26.5
	[M + ^109^Ag]^+^	26498	298.8
	[M + ^109^Ag_3_]^+^	8568	333.1
11-COOH-THC	[M + H]^+^	1102	9.3
	[M + ^109^Ag]^+^	17908	228.9
	[M + ^109^Ag_3_]^+^	3202	83.9
THC	[M + ^109^Ag]^+^	398	7.7
fentanyl	[M + H]^+^	6835	110.3
	[M + Na]^+^	830	15.2
	[M + ^109^Ag]^+^	18285	479.8
codeine	[M + H]^+^	863	18.4
	[M + Na]^+^	829	14.6
	[M + K]^+^	369	6.6
	[M + ^109^Ag]^+^	851	22.6
	[M – H]^+^	10158	190.5
6-MAM	[M + H]^+^	2154	16.4
	[M + Na]^+^	660	7.3
	[M + ^109^Ag]^+^	1912	29.4
	[M – H]^+^	8887	119.8
heroin	[M + H]^+^	1678	18.7
	[M + Na]^+^	1687	20.3
	[M + ^109^Ag]^+^	7241	124.2
	[M – H]^+^	8286	120.2
tramadol	[M + H]^+^	692	7.2
	[M + ^109^Ag]^+^	5452	101.7
	[M – H]^+^	11161	151.4
methadone	[M + H]^+^	2894	32.0

In this study, the applied analytical method allowed for rapid screening for fentanyl and other illicit drugs. The sample preparation for ^109^AgNP-assisted LDI MS is a matter of minutes, with the spectral data acquisition itself taking even less time.^16−18^ Recently, our research has successfully applied this technique for both qualitative and quantitative analysis of low molecular weight compounds, including amino acids,^17−19^ carboxylic acids,^20−24^ and hormones.^25^ Furthermore, this method has shown exceptional utility in analyzing microorganisms^26−29^ and various human biological samples such as serum,^30^ urine,^30,31^ and tissue. Notably, we have showcased its significant potential in mass spectrometry imaging (MSI), particularly in creating detailed molecular imprints of plant^32,33^ and human tissues.^34−36^ The resulting limit of detection for fentanyl acquired using silver-109-assisted LDI MS analysis illustrates the advantages of the method for the identification and quantification of fentanyl.

## 2 Materials and Methods

### 2.1 Materials

All drugs were obtained from Sigma-Aldrich. The steel target was machined from H17 (1.4016) stainless steel. The steel target was cleaned by soaking in boiling solvents: toluene (3 × 100 mL for 30 s), chloroform (3 × 100 mL for 30 s), acetonitrile (3 × 100 mL for 30 s), and deionized water (3 × 100 mL for 30 s) before further use. The plate was dried in a high vacuum (ca. 0.01 mbar, 24 h). Solvents in use were of LC-MS grade, apart from water (18 MΩ·cm water produced locally).

### 2.2 PFL 2D GS Laser-Generated Nanomaterial (LGN) of Silver-109

Silver-109 foil used to produce nanoparticles (∼1 mm thick, 99.7% isotopic purity) was bought from Trace Sciences International (USA). The foil was placed in a glass vessel and covered by acetonitrile, with a total solvent volume of 3 mL.

Laser ablation was conducted with a 1064 nm pulsed fiber laser (Raycus RFL-P20QE/A3). The ^109^AgNPs suspension was prepared by 2 min of irradiation, with pulse energy of 0.8 mJ (100 ns pulse length) at a repetition rate of 40 kHz and scanning speed of 2000 mm/s. The ablation area was 4 × 4 mm. Suspension was transferred into a syringe immediately after ablation and used in the nebulization step.

### 2.3 Nebulization of ^109^AgNPs Suspension

The nebulization process was controlled by dedicated software. The H17 steel plate was placed on the table of a translation system consisting of a motorized XY table powered by closed-loop servomotors. A glass syringe (1 mL) was filled with the suspension of silver-109 nanoparticles prepared earlier and placed in a syringe pump (250 μL/min). The 2D system table was directed by custom-made software with 10 mm/s translation speed using a sequence of movements prepared to uniformly cover a target plate. The nebulizer was a typical, standard flow Bruker ion source nebulizer. Argon at a pressure of 2 bar was used as the nebulizing gas. Studied standards were placed on the target plate before nebulization with an automatic pipet.

### 2.4 Standard Sample Preparation

Each compound standard was diluted with methanol to give a final concentration of 100 μg/mL. Lower concentrations were prepared by further dilution of ten-times higher concentration ones. Volumes of 0.5 μL (^109^AgNPs) of selected drug solutions were placed directly on the target plate. After air drying, the target was covered with ^109^AgNPs suspension as stated previously.

### 2.5 Human Sample Preparation

The serum and urine samples were obtained from a healthy volunteer during a medical examination conducted at Hospital John Paul II in Kolbuszowa, Poland, with the purpose of detecting urinary tract malignancies. The research project received approval from the Local Bioethics Committee of the University of Rzeszów (Poland), with permit number 2018/04/10. The study was carried out in compliance with relevant rules and legislation. A volume of approximately 2.6 mL of blood was obtained from the patient and thereafter subjected to centrifugation at a speed of 3000 rpm for a duration of 10 min at ambient temperature. Blood workup was performed in the hospital and by a legally entitled medical professional working in the hospital. The serum was subsequently isolated and preserved at a temperature of −80 °C until it was needed again. Blood serum and urine were diluted 500-times with ultrapure water, and then 100 μL of serum or urine or an equal volume of previously prepared fentanyl standard solutions in concentrations from 100 μg/mL to 10 pg/mL was added, resulting in concentrations of fentanyl in spiked samples from 50 μg/mL to 5 pg/mL. The obtained samples were placed directly on a steel target plate (0.5 μL per spot) previously coated with ^109^AgNPs.

### 2.6 LDI Mass Spectrometry

Laser desorption/ionization–time-of-flight (LDI-ToF) mass spectrometry experiments were performed in reflectron mode using a Bruker Autoflex Speed time-of-flight mass spectrometer equipped with a SmartBeam II laser (352 nm). Laser impulse energy was approximately 100 μJ, and the laser repetition rate was 1000 Hz. The total number of laser shots was 9000 for each spot. This amount of laser shots was divided into three, the points positioned at a distance of ca. 1/3 of the spot radius from its center. At each point, 3000 laser shots were made with default random walk applied. The measurement range was *m*/*z* 80–1000. Suppression was turned on typically for ions of *m*/*z* lower than 80. Reflector voltages used were 21 kV (the first) and 9.55 kV (the second). The data were calibrated and analyzed with FlexAnalysis (version 3.3) using a centroid calibration model. Mass calibration was performed using internal standards (silver-109 ions and clusters from ^109^Ag^+^ to ^109^Ag_9_^+^).

## 3 Results and Discussion

**Figure 1:**
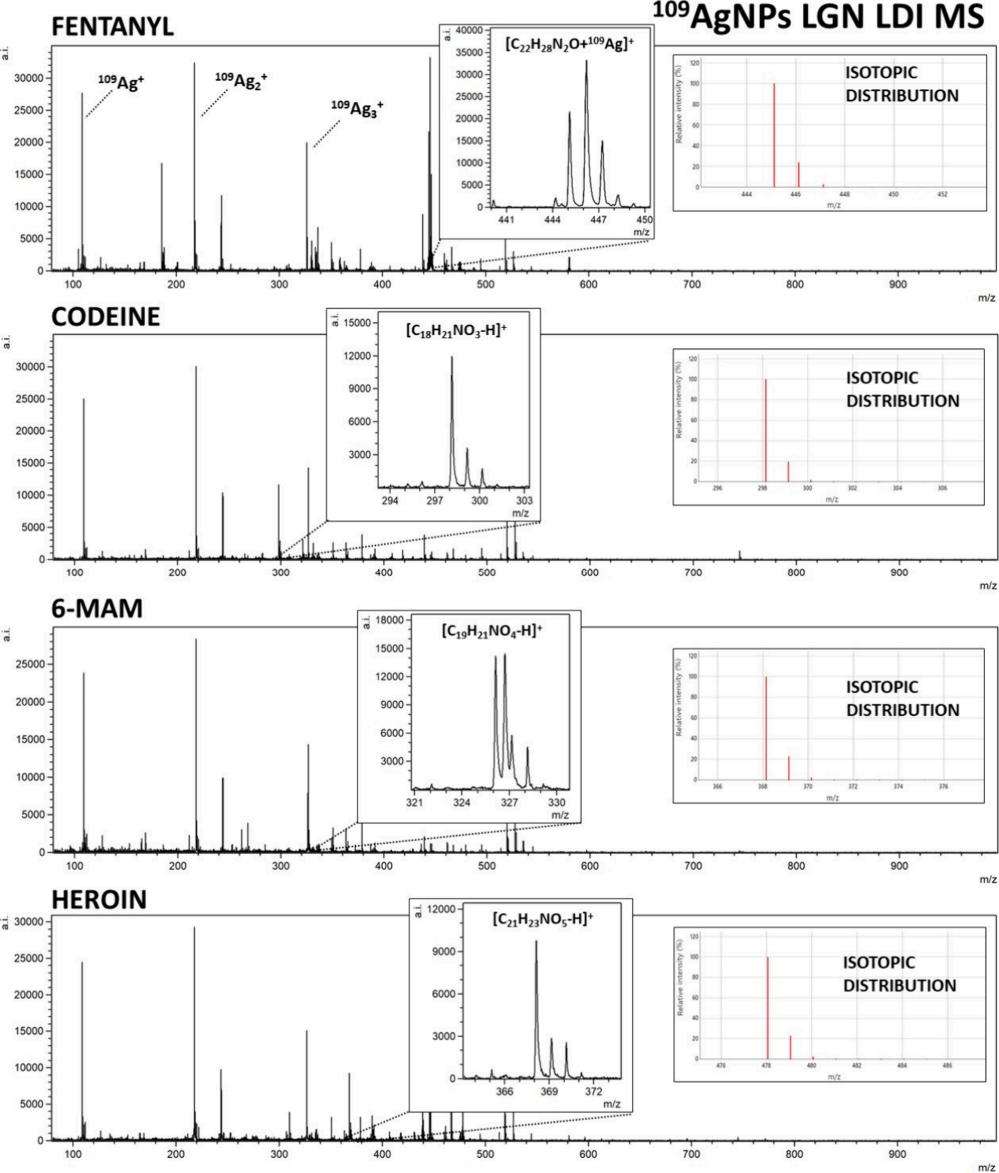
Mass spectra of opioids: fentanyl, codeine, 6-monoacetylmorphine (6-MAM), and heroin, obtained by ^109^Ag-assisted LDI MS. The spectra correspond to a sample concentration of 100 μg/mL, equating to 50 ng of compound per measurement spot. Selected identified ions and their calculated isotopic distributions are presented on the spectra.

**Figure 2:**
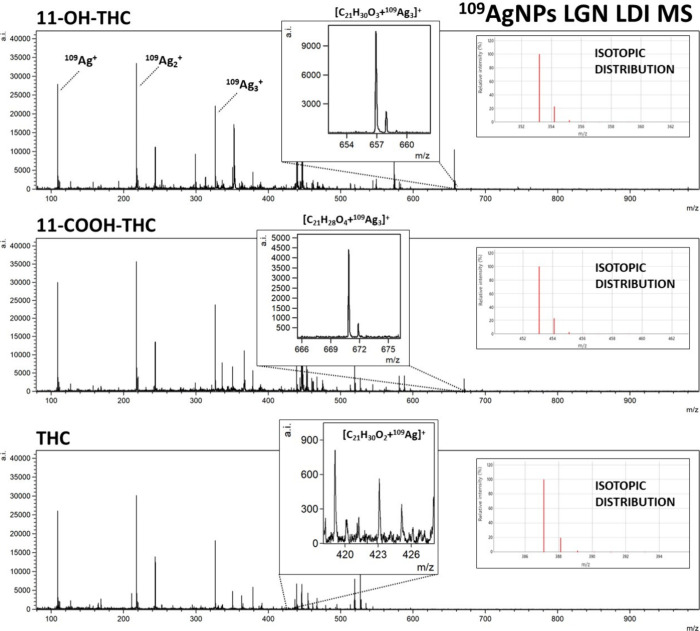
Mass spectra of THC and its metabolites, 11-OH-THC, and 11-COOH-THC, obtained by ^109^Ag-assisted LDI MS. The spectra correspond to a sample concentration of 100 μg/mL, equating to 50 ng of compound per measurement spot. Selected identified ions and their isotopic distributions are presented on the spectra.

**Figure 3:**
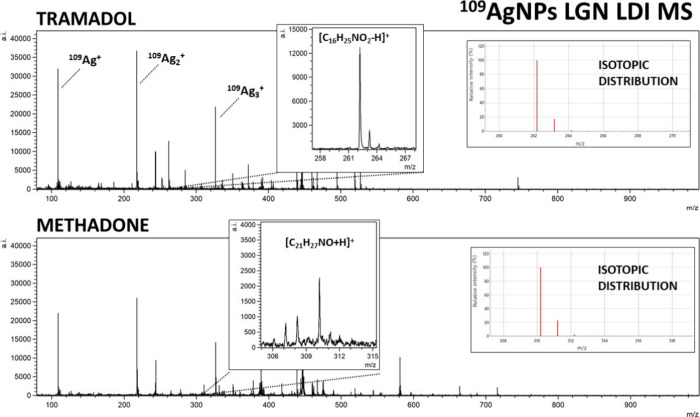
Mass spectra of tramadol and methadone obtained by ^109^Ag-assisted LDI MS. The spectra correspond to a sample concentration of 100 μg/mL, equating to 50 ng of compound per measurement spot. Selected identified ions and their isotopic distributions are presented on the spectra.

In this research, the applicability of ^109^AgNPs-assisted laser desorption/ionization MS for identification and quantification of fentanyl and other drugs, such as THC and its two metabolites, as well as opioids, including tramadol, 6-monoacetylmorphine (6-MAM), codeine, methadone, and heroin, was presented. Silver-109 nanoparticle-enhanced LDI applicability for samples of various compositions has been presented by our team in the past.^19,21,25^ Obtained mass spectra are presented in Figures 1–3. The list of identified ions with the average signal intensity and signal-to-noise ratio of the analyzed compounds at a concentration of 100 μg/mL is presented in Table 1.

For all of the analyzed opioids, except methadone, [M + ^109^Ag]^+^ ions were identified in corresponding spectra, which further confirms the validity of silver-109-nanoparticle-assisted LDI as an effective analytical method. Especially high signal intensity has been observed for [fentanyl + ^109^Ag]^+^ adduct ions, as well as for [11-OH-THC + ^109^Ag]^+^ (Figure 1) and [11-COOH-THC + ^109^Ag]^+^ (Figure 2). Nonetheless, the mass spectra of codeine, heroin, 6-MAM, and tramadol allowed for the identification of [M – H]^+^ ions of significantly higher intensities than [M + ^109^Ag]^+^ ions of the given sample (Figures 1 and 3). The formation of deprotonated ions with positive charge is characteristic for tertiary amines. It is believed to be a result of photoionization caused by the UV laser used in the MALDI MS ion source. The mechanism of this process is explained as the initial protonation of the N atom, followed by deprotonation and formation of the C=N double bond.^37,38^

For THC and its metabolites, 11-OH-THC and 11-COOH-THC, the most prominent identified ion was [M + ^109^Ag]^+^, with [M + H]^+^ (for OH-THC and COOH-THC) and [M + Na]^+^ (for OH-THC) also identifiable (Figure 2). Peaks of significant intensity corresponding to rarely appearing triatomic silver-109 cation adducts [M + ^109^Ag_3_]^+^ were also present for the two THC metabolites. Mentioned adducts have been recorded previously for amino acids^39^ and other compounds.^40^ While a distinct occurrence of those adducts has been documented previously for codeine,^41^ the [codeine + ^109^Ag_3_]^+^ peak on the mass spectrum acquired in this research was not of significant intensity. An assumption can be made that the occurrence of [M + ^109^Ag_3_]^+^ ions has little correlation with the nature of the compound, rather depending on the conditions of the analysis. Sodium and potassium adducts were also identified for selected samples as presented in Table 1.

### 3.1 Quantification Results

The measurements of analyzed standards were performed in the concentration range from 100 μg/mL to 10 pg/mL. Samples were placed on the target plate in a 0.5 μL volume equating to 50 ng to 50 fg of compound per spot. The limit of detection (LOD) was calculated based on the S/N ratio of the highest intensity adduct monoisotopic signal of 3, obtained from the mass spectra of the lowest concentration from each sample. The lower limit of quantification (LLOQ) was calculated using an S/N ratio of 10. A regression analysis of mean intensity as a function of sample concentration was conducted. The LOD, LLOQ results, and *R*^2^ values for the linear regressions of the tested compounds are presented in Table 2.

**Table 2:** Limits of Detection (LOD), Limits of Quantification (LOQ), and Linearity *R*^2^ for Tested Compounds

compound	LOD	LLOQ	*R*^2^
11-OH-THC	538.92 ± 207.47 ng/mL	1796.41 ± 691.57 ng/mL	0.97
11-COOH-THC	6671.61 ± 202.18 ng/mL	22238.7 ± 6739.38 ng/mL	0.96
THC	39.13 ± 20.76 μg/mL	130.43 ± 69.23 μg/mL	0.98
fentanyl	1.03 ± 0.19 pg/mL	3.43 ± 0.66 pg/mL	0.98
codeine	61.22 ± 8.7 ng/mL	204.08 ± 29.01 ng/mL	0.94
6-MAM	57.14 ± 10.16 ng/mL	190.48 ± 33.87 ng/mL	0.97
heroin	8.22 ± 0.16 ng/mL	27.4 ± 0.53 ng/mL	0.86
tramadol	44.55 ± 29.98 ng/mL	148.51 ± 99.92 ng/mL	0.99
methadone	386.27 ± 8.93 ng/mL	1287.55 ± 297.51 ng/mL	0.96

**Figure 4:**
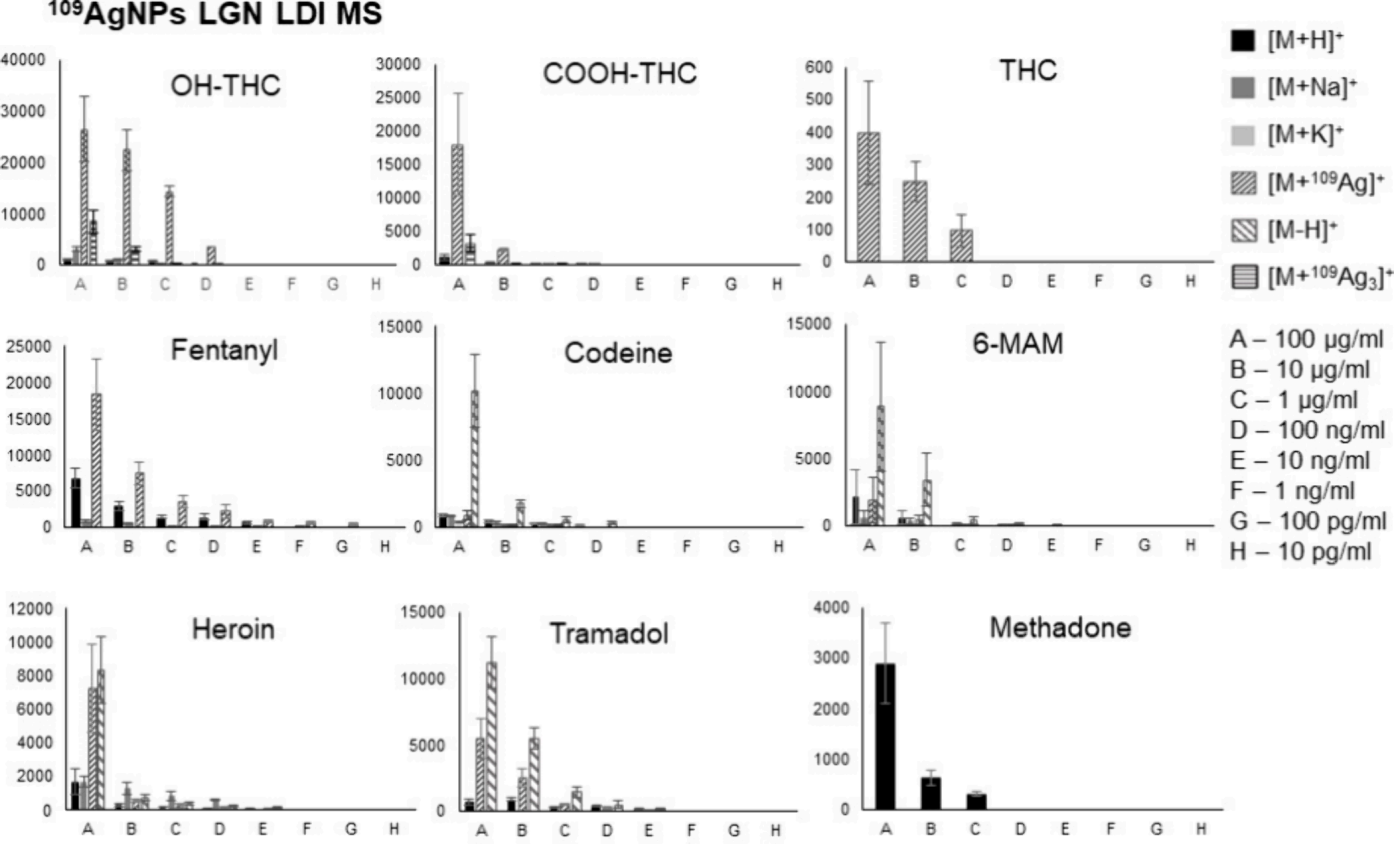
Relation of the mean intensity of adduct ions versus sample concentration, along with standard derivation for each measuring spot presented as bar charts for all tested compounds.

Figure 4 presents the relations of all identified adduct ions’ signal intensities against the concentration of the compound in the sample. For Δ^9^-THC, the average signal intensity values were rather low, which might cause the quantification of said compound in lower concentrations to be unattainable. Nevertheless, both THC metabolites showed sufficient signal intensity, which suggests that the method might be suitable for the detection of THC-containing drug usage in humans in connection with the reasons stated previously. The results for most of the analyzed standards indicate that using ^109^AgLGN for LDI MS has its usefulness in illicit drug identification and quantification within the used sample concentration range, making it suitable for regression analysis.

**Figure 5:**
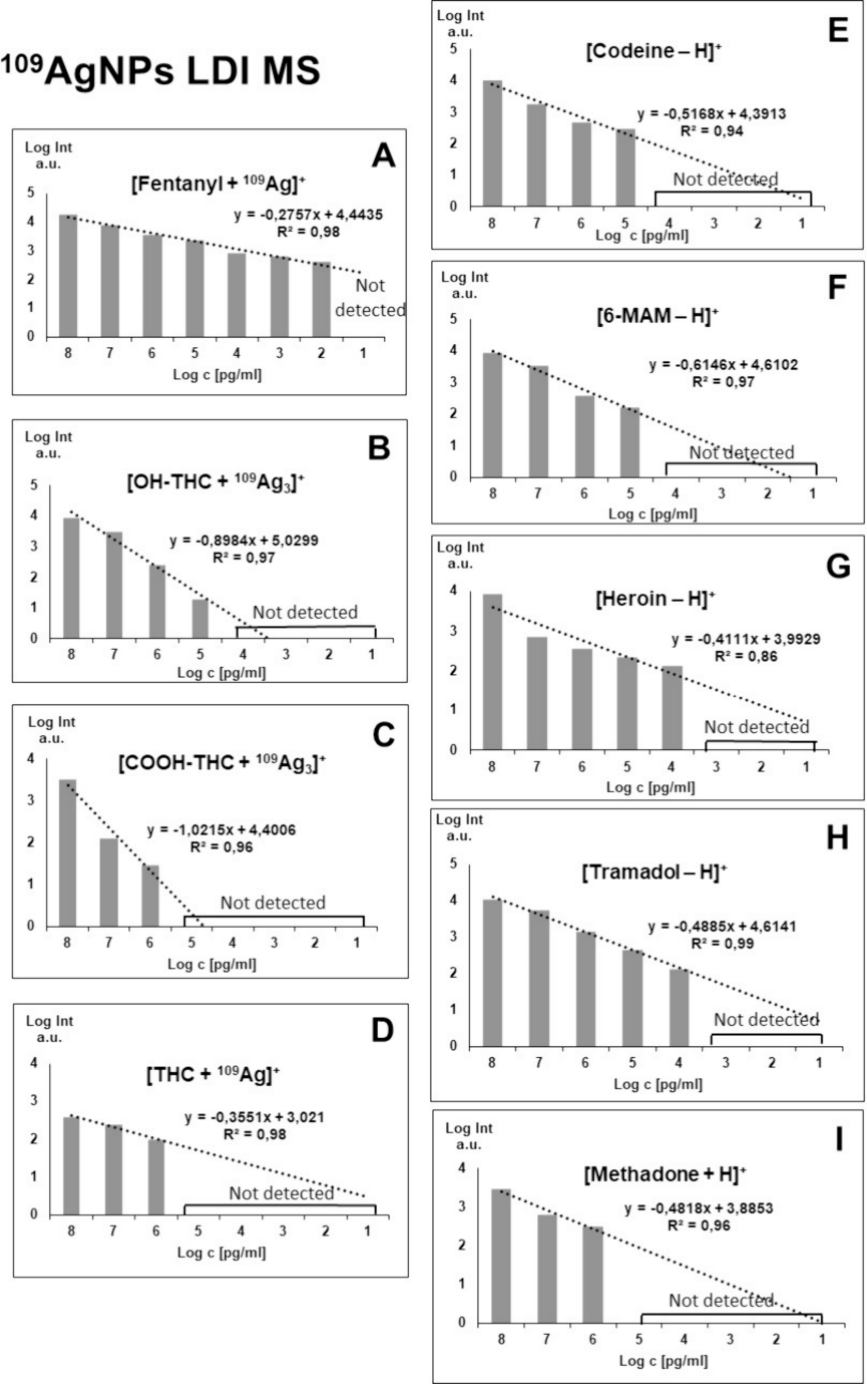
Bar charts presented for the relation of logarithm of signal intensity against the logarithm of concentration, along with regression lines and equations for A: fentanyl, B: 11-OH-THC, C: 11-COOH-THC, D: Δ^9^-THC, E: codeine, F: 6-monoacetylmorphine, G: heroin, H: tramadol, I: methadone.

**Figure 6:**
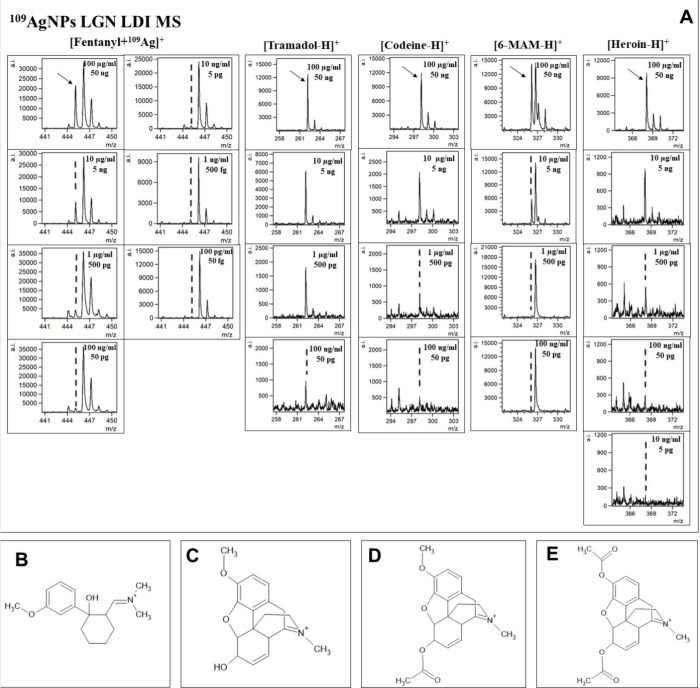
A: Spectra fragments for selected ions of interest presented for different concentrations of fentanyl, tramadol, codeine, 6-MAM, and heroin, with corresponding concentrations and compound mass per spot presented. Proposed structures of uncommon deprotonated ions: B, [tramadol – H]^+^; C, [codeine – H]^+^; D, [6-MAM – H]^+^; E, [heroin – H]^+^.

**Figure 7:**
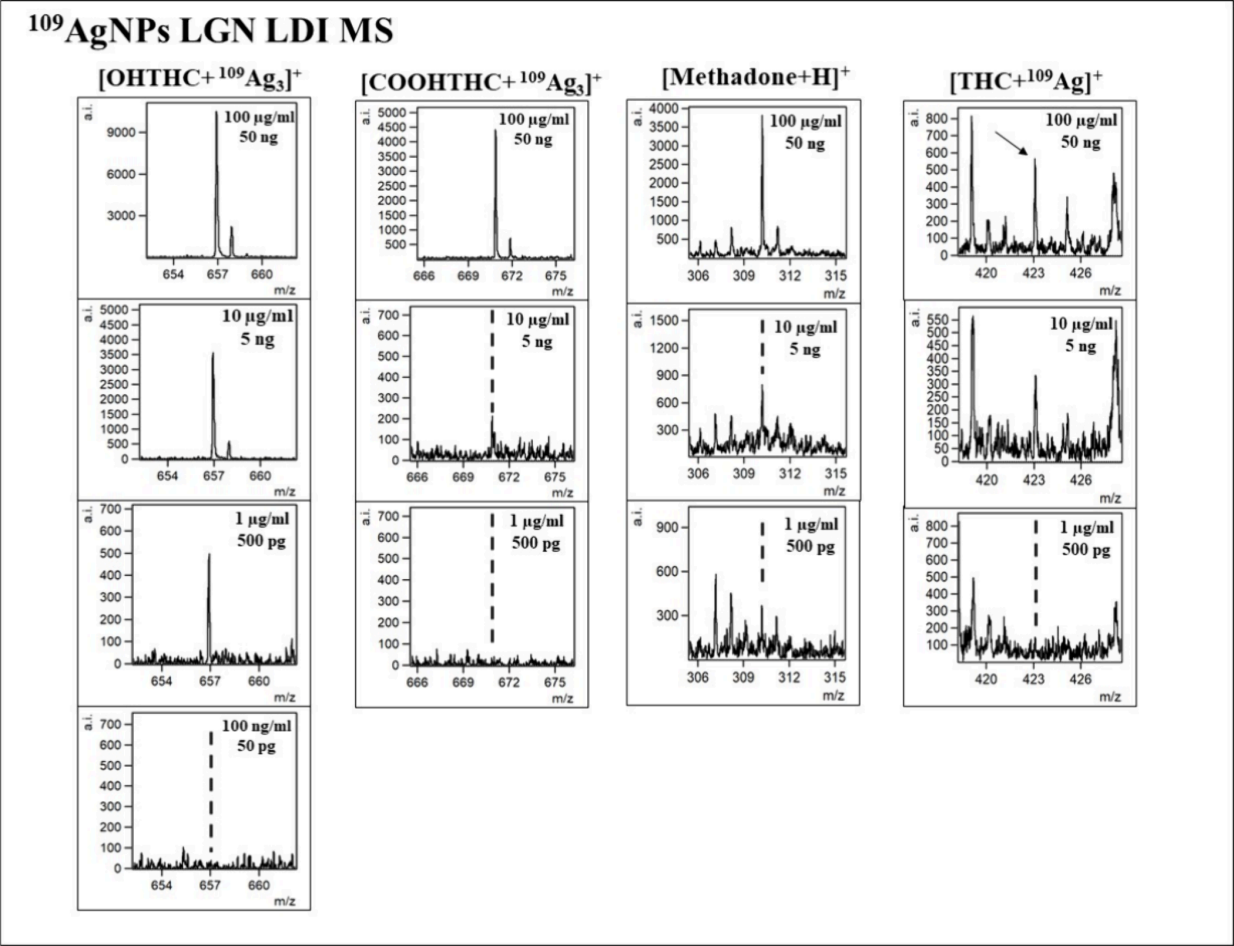
Spectra fragments for selected ions of interest presented for lowering concentrations for 11-OH-THC, 11-COOH-THC, methadone, and Δ^9^-THC with corresponding concentrations and compound weight per spot presented.

Figure 5 shows bar charts with corresponding linear regression functions. In the case of OH-THC and COOH-THC the [M + ^109^Ag_3_]^+^ adducts were chosen for analysis; for THC and fentanyl, the [M + ^109^Ag]^+^ ions were used, for methadone, [M + H]^+^ was the selected adduct ion, and for codeine, 6-MAM, heroin, and tramadol the [M – H]^+^ ions were the most suitable. Additionally, Figures 6 and 7 present the selected ions of each analyzed compound, showcasing the decrease in signal intensity.

The first analyzed opioid was fentanyl, a synthetic analgesic. Despite structurally having traits of a tertiary amine, no significant signal intensities of [M – H]^+^ ion were observed for said compound. Regardless of that, an exceptional result was obtained for the [C_22_H_28_N_2_O + ^109^Ag]^+^ adduct ion. A logarithmic relationship between intensity and concentration was found for almost all analyzed concentrations, except the lowest one, in a range of 100 μg/mL to 100 pg/mL. LOD value was found to be 1.03 pg/mL (0.515 fg/per spot). The coefficient of determination found for acquired data was 0.98 (Figure 5 A). In the work of Angi et al. from 2019,^42^ the UHPLC method coupled with single quadrupole mass spectrometry was used to determine 19 fentanyl analogues also using standard solutions diluted with methanol. The authors managed to obtain an LOD of 3 ng/mL for fentanyl. The LOD in this current research is approximately 3000 times lower than that reported in the LC-MS method, suggesting that ^109^AgNP-assisted LDI MS is a powerful method of screening for fentanyl.

The 11-OH-THC adduct ion [C_21_H_30_O_3_ + ^109^Ag_3_]^+^ was identified in the four highest concentrations, from 100 μg/mL to 100 ng/mL. LOD was calculated to be 0.54 μg/mL (270 pg/per spot). As presented in Figure 5B, the linear regression function gave the coefficient of determination *R*^2^ value of 0.97. For 11-COOH-THC, the triatomic silver cation adduct was also chosen for the regression analysis. The [C_21_H_28_O_4_ + ^109^Ag_3_]^+^ ion was found in the three highest sample concentrations, from 100 μg/mL to 1 μg/mL. LOD was found to be 6.67 μg/mL (3.34 ng/per spot). The *R*^2^ value calculated by the regression analysis of this compound is 0.96 as seen in Figure 5B. For Δ^9^-THC, only [C_21_H_30_O_2_ + ^109^Ag]^+^ adduct ion has shown the logarithmic relationship of intensity on concentration. It was found in the three highest concentrations. The LOD value was calculated to be 39.13 μg/mL (19.57 ng/per spot) and the *R*^2^ value was determined as 0.98. Referring to the previously mentioned study from Heo et al.,^43^ the LOD and LOQ determined for THC were respectively 0.6 ng/mL and 0.0018 in methanol-diluted standard samples using UPLC–MS/MS with a triple quadrupole detector. The corresponding coefficient of determination was 0.9999. In the research conducted by Simões et al.,^44^ the LOD values obtained from the calibration curve for standard solutions dissolved in methanol were 0.5 ng/mL for 11-OH-THC and THC-COOH and 0.5 ng/mL for THC.

Codeine, naturally found in opium poppy produced the highest intensity ion assigned to [C_18_H_21_NO_3_ – H]^+^ formula. It was identified as the four highest concentrations. The limit of detection calculated for codeine was 61.22 ng/mL (30.6 pg/per spot). The *R*^2^ value was found to be 0.94. For 6-monoacetylmorphine, which is a metabolite of heroin, a logarithmic relationship of intensity versus concentration was found for the 100 μg/mL to 100 ng/mL range. The ion used for regression analysis was [C_19_H_21_NO_4_ – H]^+^. LOD calculated by using the signal-to-noise ratio was found to be 57.14 ng/mL (28.57 pg/per spot). The bar chart used for the analysis of regression is presented in Figure 4F, with an *R*^2^ value of 0.97. In research conducted in 2016 by Gul et al.^45^ regarding the analysis of opiates in wastewater using LC-MS/MS, the LOD and LOQ values determined for standard solutions of codeine and 6-MAM were 0.62 ng/mL with an *R*^2^ coefficient of 0.9998.

The following analyzed compound was heroin, a semisynthetic opioid. The [C_21_H_23_NO_5_ – H]^+^ ion was found in the five highest concentrations from 100 μg/mL to 10 ng/mL Limit of detection value for heroin was found to be 8.22 ng/mL (4.11 pg/per spot). As seen in Figure 5G, the coefficient of determination *R*^2^ was 0.86. In a study conducted in 2020 by Jovanov et al.,^46^ which presented a rapid method for detecting heroin using HPLC–MS/MS, an LOD and LOQ were obtained from calibration curves of a 0.4 μg/mL heroin standard solution.

The regression analysis of tramadol, a synthetic opioid produced as pain medication, was also based on its [M – H]^+^ ion. Signals of significant intensity were found in the five highest concentrations, as presented in Figure 5H. LOD was calculated and found to be 44.55 ng/mL (22.28 pg/per spot), and the *R*^2^ coefficient was determined to be 0.99. Abdelshakour et al.^47^ conducted a study utilizing UPLC-MS/MS to quantify tramadol in methanol-diluted standard solutions. They achieved a limit of detection (LOD) of 0.015 μg/mL and a limit of quantification (LOQ) of 0.05 μg/mL for tramadol, with an *R*_2_ value of 0.9999.

The last analyzed compound was methadone, also a synthetic opioid, often used for heroin addiction treatment. For this opioid, only the [C_21_H_27_NO + H]^+^ ion has shown linear regression in terms of intensity and concentration relationship, as presented in Figure 4I. It was identified for the three highest concentrations, in the 100 μg/mL to 1 μg/mL range. The calculated LOD value was 386 ng/mL (193 pg/per spot). The determination coefficient *R*^2^ value was found to be 0.96. In research conducted by Whitehead et al.^48^ in which the LC-MS/MS method was used to detect illicit drugs, the LOD and LOQ values determined for standard solutions were 3.8 pg/mL and 0.1 ng/mL, respectively.

### 3.2 Matrix Effect

Biological samples, such as tissues or body fluids, are considered to be a complex matrix containing, among others, proteins, peptides, lipids, and other endogenous compounds that may interfere with the detection of illicit drugs. This complexity often requires additional sample preparation steps, such as protein precipitation, solid-phase extraction, or liquid–liquid extraction, to isolate drugs from the biological matrix. Moreover, matrix effects in biological samples can affect the accuracy and sensitivity of detection methods. In contrast, our study, which uses standards diluted in methanol, avoids such matrix effects and can therefore demonstrate the capabilities of the analytical method in an idealized scenario. To validate the effectiveness of ^109^AgNPs LGN LDI MS in the analysis of illicit drugs in complex mixtures such as biological samples, we conducted additional experiments on samples spiked with illicit drugs

The matrix effect was assessed by evaluating the ion intensity of fentanyl adducts. This involved comparing the intensity of ions from fentanyl in diluted urine and serum samples, which had been spiked with standard compounds, against the ion intensity of corresponding adducts in pure standard solutions diluted in methanol (Figures S1 and S2, Supporting Information). The examination of signal intensities across the entire spectrum of tested fentanyl concentrations reveals that the adduct formed with silver-109 ions ([fentanyl + ^109^Ag]^+^) was the most prevalent ion species for this compound. This is evidenced by the significantly stronger signals for the silver adduct in comparison to those of the sodium ion adducts ([fentanyl + Na]^+^) and the protonated fentanyl ions ([fentanyl + H]^+^). The prominence of the silver adduct ion was consistent not only in the standard methanol solution but also in the diluted serum and urine samples. The LOD data indicate a high degree of method sensitivity for detecting fentanyl, both in the standard solution and in serum samples spiked with fentanyl, evidenced by LODs of 3.36 ± 0.68 pg/mL and 3.04 ± 0.81 pg/mL, respectively. For spiked urine, LOD was 4.05 ± 0.76 pg/mL. These surprisingly good results are probably results of 1000-times dilution of serum and urine, dilution that is needed in our method mainly due to the formation of thick films of material on the target plate. In this case it would be proper to recalculate LODs to include matrix dilution which gives 3.04 ± 0.81 and 4.05 ± 0.76 ng/mL for serum and urine, respectively. While biological matrix results are comparable to the ones from LCMS studies, still fentanyl LOD for standard solution in methanol is among the lowest to date. Previously, Zhang et al. achieved one of the lowest reported LOD for fentanyl, reaching 2.5 ng/mL in diluted urine samples and 0.5 ng/mL in diluted blood samples, using a liquid chromatography–high resolution mass spectrometry.^49^ Meanwhile, Busardo et al. recorded LOD values in the picogram per milliliter range for fentanyl, but with a lower resolution triple quadrupole mass spectrometer.^50^ However, both studies involved complicated and time-consuming sample preparation processes, including protein precipitation, that were considerably more time-intensive compared to our rapid method.

### 3.3 Advantages and Disadvantages of ^109^AgNPs LGN LDI MS

The laser desorption/ionization mass spectrometry method using silver-109 nanoparticles (^109^AgNPs-LDI-MS) we’ve introduced offers considerable advantages over the commonly used atmospheric pressure ionization techniques in toxicological analyses and the detection of new psychoactive substances. A primary benefit of ^109^AgNPs LGN LDI MS is its capacity for rapid analysis: up to 100 samples on a single 4.5 × 3.5 cm plate; the spectrum is ready typically within seconds per sample, with the potential for automation. This capability significantly enhances throughput. Unlike LC-MS methods, ^109^AgNPs-LDI-MS is much less affected by matrix effects, reducing sample preparation time. For small molecular analyses in complex biological matrices such as blood or urine, a simple dilution suffices, eliminating the need for elaborate extraction and protein precipitation. While LC-MS often struggles with matrix effects leading to variable reproducibility, ^109^AgNPs LGN LDI MS achieves superior sensitivity compared to other high-resolution methods under atmospheric pressure. On the other hand, high-performance liquid chromatography coupled with high-resolution mass spectrometry (UHPLC-HRMS) offers a robust advantage for the comprehensive and selective analysis of complex biological mixtures. By separating sample components on a chromatographic column before mass spectrometric analysis, UHPLC-HRMS is essential for the precise identification and quantification of chemical compounds within samples of complex composition. However, the LC-MS method is notably vulnerable to sample contamination; depending on the nature of the sample, contaminants could be introduced, potentially leading to the blockage of columns or tubing. Furthermore, the process of optimizing experimental conditions for LC-MS can be time-intensive, posing challenges when rapid analysis of numerous samples with minute analyte quantities is required. Additionally, sample availability often constrains LC-MS, particularly when the analyte of interest is unidentified and occurs in small amounts in the sample, making it challenging to ascertain the appropriateness of a specific dilution for detection.

## 4 Conclusion

LDI MS with the application of laser-generated silver-109-nanoparticles enabled the detection and quantification of illicit drugs in a broad range of concentrations, from 100 μg/mL to 100 pg/mL in the case of fentanyl. Analyzed standards produced typical adduct ions such as protonated, sodiated ions, and also ions specific to this method, adducts of ^109^Ag^+^, and also less known [M + ^109^Ag_3_]^+^ and uncommon [M – H]^+^ ions. The chosen method turned out to be most suitable for rapid screening for fentanyl, resulting in a low limit of detection, giving exceptional results compared to other published reports. It is therefore a suitable analytical method for the detection and quantification of illicit drug occurrence and use.

## Data Availability

The data sets generated during and/or analyzed during the current study are available from the corresponding author upon request and in the RepOD open data repository (doi: 10.18150/EJLW9K).

## Supporting Information Available

Bar graphs showing the relationship between the log intensity of adduct ions and the log concentration of fentanyl in various samples: a methanol standard and diluted serum and urine. Spectra fragments displaying fentanyl ion signals in a standard solution and in fentanyl-spiked diluted serum and urine (PDF)
