# Supplementary material for: Fiber Laser-Generated Silver-109 Nanoparticles for Laser Desorption/Ionization Mass Spectrometry of Illicit Drugs
Source: J Am Soc Mass Spectrom. 2024 May 6;35(6):1156–67. doi: 10.1021/jasms.3c00454 (PMC11157659; doi:10.1021/jasms.3c00454)
Supplement: Supplementary file 1 — js3c00454_si_001.pdf [file js3c00454_si_001.pdf]

Supplementary information

Fiber laser-generated silver-109 nanoparticles for laser desorption/ionization mass spectrometry of illicit drugs

Zuzanna Krupa[1,2] and Joanna Nizioł[2*]

[1]*Doctoral School of Engineering and Technical Sciences at the Rzeszów University of Technology, 8 Powstańców Warszawy Ave., 35-959 Rzeszów, Poland.*
[2]*Rzeszów University of Technology, Faculty of Chemistry, 6 Powstańców Warszawy Ave., 35-959 Rzeszów, Poland.*

*Corresponding author: Joanna Nizioł, e-mail: jniziol@prz.edu.pl, tel: (+48 17) 865-1310

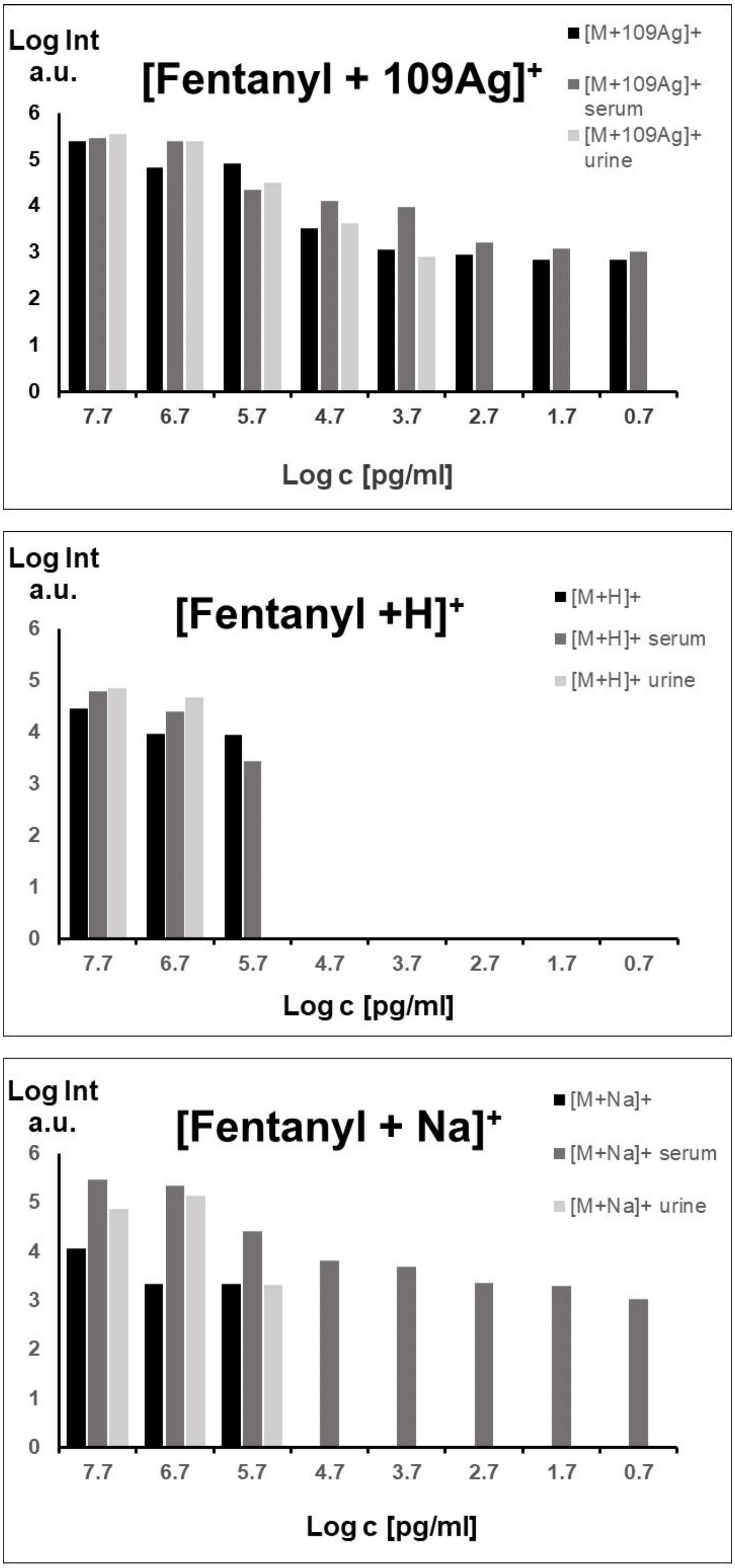

Figure S1. Bar graphs illustrating the relation between logarithm of intensity of adduct ions in analyzed samples: fentanyl standard in methanol, fentanyl in diluted serum, fentanyl in diluted urine *versus* the logarithm of concentration of fentanyl in each sample.

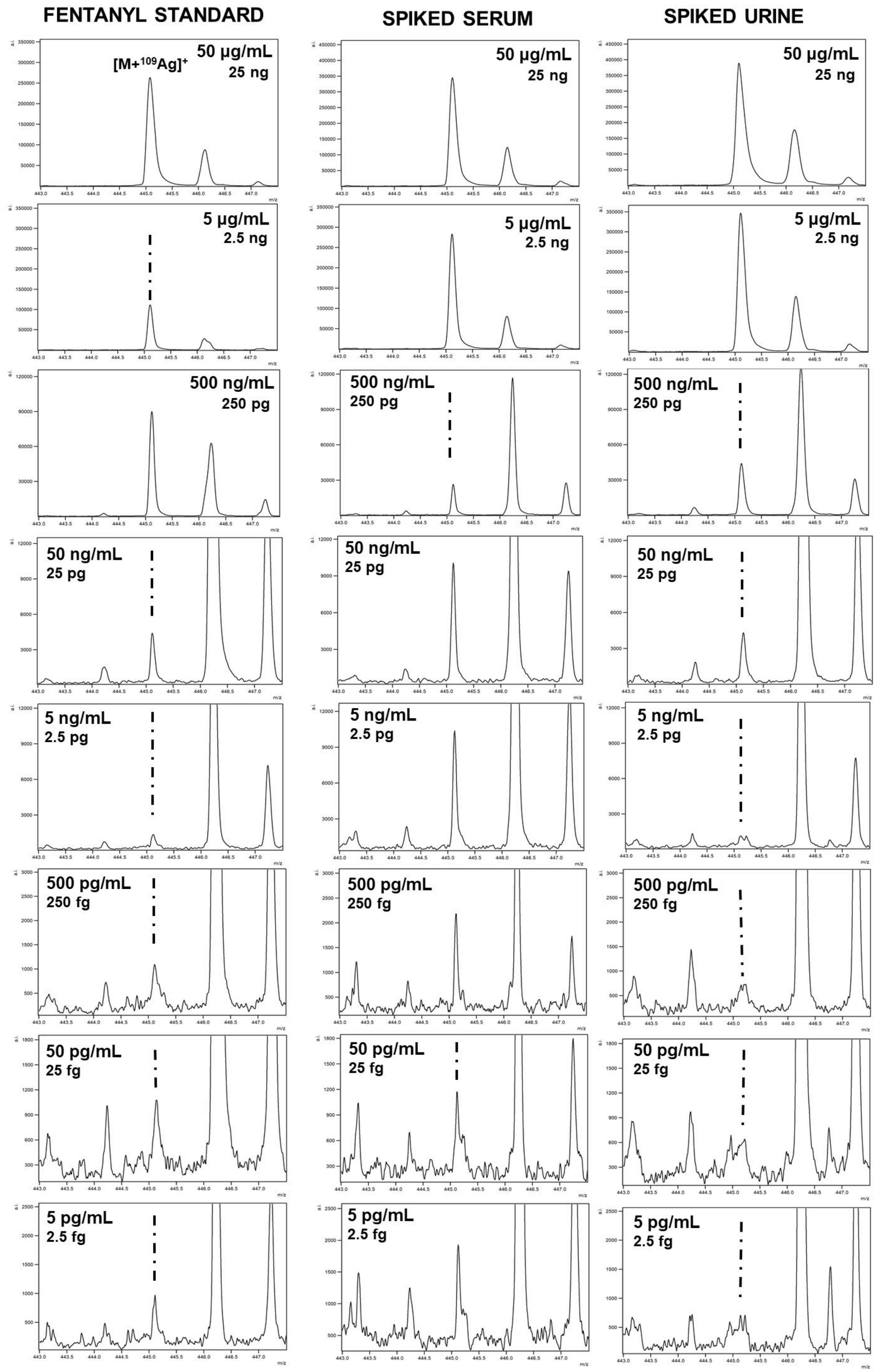

Figure S2. Spectra fragments containing [fentanyl+^{109}Ag]$^{+}$ ion signals for standard solution (left column), diluted serum spiked with fentanyl standard (center column) and diluted urine spiked with fentanyl standard.
